# Supplementary material for: Unveiling the Bioactive Potential of the Invasive Jellyfish Phyllorhiza punctata Through Integrative Transcriptomic and Proteomic Analyses
Source: Biomolecules. 2025 Aug 4;15(8):1121. doi: 10.3390/biom15081121 (PMC12383608; doi:10.3390/biom15081121)
Supplement: Supplementary file 1 [file biomolecules-15-01121-s001.zip › Suplementary Material/FileS3/PEPFOLD4/XP_065068687_15073/clusters.html]

 PEP-FOLD clusters


Download archive of all models  here   

|  |  |  |
| --- | --- | --- |
| # | file | sOPEP |
| # cluster 1 - Size: 7 |  |  |
|  | XP\_065068687\_15073-model1 | -23.499 |
|  | XP\_065068687\_15073-model1.1 | -23.3774 |
|  | XP\_065068687\_15073-model1.2 | -22.8457 |
|  | XP\_065068687\_15073-model1.3 | -22.469 |
|  | XP\_065068687\_15073-model1.4 | -14.9801 |
|  | XP\_065068687\_15073-model1.5 | -14.4326 |
|  | XP\_065068687\_15073-model1.6 | -11.3552 |
| # cluster 2 - Size: 5 |  |  |
|  | XP\_065068687\_15073-model2 | -23.2416 |
|  | XP\_065068687\_15073-model2.1 | -17.535 |
|  | XP\_065068687\_15073-model2.2 | -16.6354 |
|  | XP\_065068687\_15073-model2.3 | -15.3564 |
|  | XP\_065068687\_15073-model2.4 | -14.9624 |
| # cluster 3 - Size: 2 |  |  |
|  | XP\_065068687\_15073-model3 | -22.4878 |
|  | XP\_065068687\_15073-model3.1 | -12.7248 |
| # cluster 4 - Size: 1 |  |  |
|  | XP\_065068687\_15073-model4 | -22.1819 |
| # cluster 5 - Size: 1 |  |  |
|  | XP\_065068687\_15073-model5 | -21.604 |
| # cluster 6 - Size: 1 |  |  |
|  | XP\_065068687\_15073-model6 | -20.7033 |
| # cluster 7 - Size: 3 |  |  |
|  | XP\_065068687\_15073-model7 | -20.6708 |
|  | XP\_065068687\_15073-model7.1 | -18.7571 |
|  | XP\_065068687\_15073-model7.2 | -17.1283 |
| # cluster 8 - Size: 1 |  |  |
|  | XP\_065068687\_15073-model8 | -20.2993 |
| # cluster 9 - Size: 1 |  |  |
|  | XP\_065068687\_15073-model9 | -19.7967 |
| # cluster 10 - Size: 1 |  |  |
|  | XP\_065068687\_15073-model10 | -19.6755 |
| # cluster 11 - Size: 1 |  |  |
|  | XP\_065068687\_15073-model11 | -19.6305 |
| # cluster 12 - Size: 4 |  |  |
|  | XP\_065068687\_15073-model12 | -19.5822 |
|  | XP\_065068687\_15073-model12.1 | -18.6906 |
|  | XP\_065068687\_15073-model12.2 | -17.054 |
|  | XP\_065068687\_15073-model12.3 | -11.0141 |
| # cluster 13 - Size: 1 |  |  |
|  | XP\_065068687\_15073-model13 | -19.3282 |
| # cluster 14 - Size: 2 |  |  |
|  | XP\_065068687\_15073-model14 | -19.2499 |
|  | XP\_065068687\_15073-model14.1 | -16.6305 |
| # cluster 15 - Size: 2 |  |  |
|  | XP\_065068687\_15073-model15 | -19.1348 |
|  | XP\_065068687\_15073-model15.1 | -12.878 |
| # cluster 16 - Size: 1 |  |  |
|  | XP\_065068687\_15073-model16 | -19.075 |
| # cluster 17 - Size: 2 |  |  |
|  | XP\_065068687\_15073-model17 | -18.8324 |
|  | XP\_065068687\_15073-model17.1 | -17.679 |
| # cluster 18 - Size: 1 |  |  |
|  | XP\_065068687\_15073-model18 | -18.4995 |
| # cluster 19 - Size: 6 |  |  |
|  | XP\_065068687\_15073-model19 | -17.9818 |
|  | XP\_065068687\_15073-model19.1 | -17.4185 |
|  | XP\_065068687\_15073-model19.2 | -16.9957 |
|  | XP\_065068687\_15073-model19.3 | -16.2874 |
|  | XP\_065068687\_15073-model19.4 | -15.5286 |
|  | XP\_065068687\_15073-model19.5 | -14.5407 |
| # cluster 20 - Size: 2 |  |  |
|  | XP\_065068687\_15073-model20 | -17.8169 |
|  | XP\_065068687\_15073-model20.1 | -13.7459 |
| # cluster 21 - Size: 2 |  |  |
|  | XP\_065068687\_15073-model21 | -17.7612 |
|  | XP\_065068687\_15073-model21.1 | -16.6949 |
| # cluster 22 - Size: 2 |  |  |
|  | XP\_065068687\_15073-model22 | -17.4471 |
|  | XP\_065068687\_15073-model22.1 | -14.7478 |
| # cluster 23 - Size: 2 |  |  |
|  | XP\_065068687\_15073-model23 | -17.2671 |
|  | XP\_065068687\_15073-model23.1 | -14.1037 |
| # cluster 24 - Size: 1 |  |  |
|  | XP\_065068687\_15073-model24 | -16.8823 |
| # cluster 25 - Size: 5 |  |  |
|  | XP\_065068687\_15073-model25 | -16.5258 |
|  | XP\_065068687\_15073-model25.1 | -13.8559 |
|  | XP\_065068687\_15073-model25.2 | -13.8521 |
|  | XP\_065068687\_15073-model25.3 | -13.6169 |
|  | XP\_065068687\_15073-model25.4 | -13.0619 |
| # cluster 26 - Size: 1 |  |  |
|  | XP\_065068687\_15073-model26 | -16.4123 |
| # cluster 27 - Size: 1 |  |  |
|  | XP\_065068687\_15073-model27 | -16.1805 |
| # cluster 28 - Size: 1 |  |  |
|  | XP\_065068687\_15073-model28 | -16.1222 |
| # cluster 29 - Size: 2 |  |  |
|  | XP\_065068687\_15073-model29 | -16.0698 |
|  | XP\_065068687\_15073-model29.1 | -14.2213 |
| # cluster 30 - Size: 2 |  |  |
|  | XP\_065068687\_15073-model30 | -16.0471 |
|  | XP\_065068687\_15073-model30.1 | -11.7088 |
| # cluster 31 - Size: 1 |  |  |
|  | XP\_065068687\_15073-model31 | -15.7506 |
| # cluster 32 - Size: 2 |  |  |
|  | XP\_065068687\_15073-model32 | -15.6616 |
|  | XP\_065068687\_15073-model32.1 | -11.4211 |
| # cluster 33 - Size: 1 |  |  |
|  | XP\_065068687\_15073-model33 | -15.6472 |
| # cluster 34 - Size: 1 |  |  |
|  | XP\_065068687\_15073-model34 | -15.3927 |
| # cluster 35 - Size: 1 |  |  |
|  | XP\_065068687\_15073-model35 | -15.2529 |
| # cluster 36 - Size: 1 |  |  |
|  | XP\_065068687\_15073-model36 | -15.0834 |
| # cluster 37 - Size: 1 |  |  |
|  | XP\_065068687\_15073-model37 | -15.051 |
| # cluster 38 - Size: 3 |  |  |
|  | XP\_065068687\_15073-model38 | -15.017 |
|  | XP\_065068687\_15073-model38.1 | -14.7108 |
|  | XP\_065068687\_15073-model38.2 | -14.1442 |
| # cluster 39 - Size: 1 |  |  |
|  | XP\_065068687\_15073-model39 | -14.8693 |
| # cluster 40 - Size: 1 |  |  |
|  | XP\_065068687\_15073-model40 | -14.7509 |
| # cluster 41 - Size: 2 |  |  |
|  | XP\_065068687\_15073-model41 | -14.247 |
|  | XP\_065068687\_15073-model41.1 | -13.7413 |
| # cluster 42 - Size: 2 |  |  |
|  | XP\_065068687\_15073-model42 | -13.4406 |
|  | XP\_065068687\_15073-model42.1 | -12.5122 |
| # cluster 43 - Size: 1 |  |  |
|  | XP\_065068687\_15073-model43 | -13.2998 |
| # cluster 44 - Size: 1 |  |  |
|  | XP\_065068687\_15073-model44 | -13.2054 |
| # cluster 45 - Size: 1 |  |  |
|  | XP\_065068687\_15073-model45 | -13.0293 |
| # cluster 46 - Size: 2 |  |  |
|  | XP\_065068687\_15073-model46 | -13.027 |
|  | XP\_065068687\_15073-model46.1 | -10.9998 |
| # cluster 47 - Size: 1 |  |  |
|  | XP\_065068687\_15073-model47 | -12.9612 |
| # cluster 48 - Size: 1 |  |  |
|  | XP\_065068687\_15073-model48 | -12.8878 |
| # cluster 49 - Size: 1 |  |  |
|  | XP\_065068687\_15073-model49 | -12.8184 |
| # cluster 50 - Size: 1 |  |  |
|  | XP\_065068687\_15073-model50 | -12.6157 |
| # cluster 51 - Size: 1 |  |  |
|  | XP\_065068687\_15073-model51 | -12.5951 |
| # cluster 52 - Size: 1 |  |  |
|  | XP\_065068687\_15073-model52 | -12.5216 |
| # cluster 53 - Size: 1 |  |  |
|  | XP\_065068687\_15073-model53 | -12.4264 |
| # cluster 54 - Size: 1 |  |  |
|  | XP\_065068687\_15073-model54 | -12.2053 |
| # cluster 55 - Size: 1 |  |  |
|  | XP\_065068687\_15073-model55 | -12.0691 |
| # cluster 56 - Size: 1 |  |  |
|  | XP\_065068687\_15073-model56 | -11.8505 |
| # cluster 57 - Size: 1 |  |  |
|  | XP\_065068687\_15073-model57 | -11.6684 |
| # cluster 58 - Size: 1 |  |  |
|  | XP\_065068687\_15073-model58 | -11.656 |
| # cluster 59 - Size: 1 |  |  |
|  | XP\_065068687\_15073-model59 | -11.2343 |
| # cluster 60 - Size: 1 |  |  |
|  | XP\_065068687\_15073-model60 | -10.761 |
|  |  |  |
